# The Synergistic Effect of PM_2.5_ and CO_2_ Concentrations on Occupant Satisfaction and Work Productivity in a Meeting Room

**DOI:** 10.3390/ijerph18084109

**Published:** 2021-04-13

**Authors:** Jindong Wu, Jiantao Weng, Bing Xia, Yujie Zhao, Qiuji Song

**Affiliations:** 1 College of Civil Engineering and Architecture, Zhejiang University, Hangzhou 310058, China; jindongwu@zju.edu.cn (J.W.); tunitw@hotmail.com (J.W.); guan350303199@163.com (Y.Z.); 3160102915@zju.edu.cn (Q.S.); 2 Center for Balance Architecture, Zhejiang University, Hangzhou 310028, China

**Keywords:** PM_2.5_ and CO_2_ concentration, occupant satisfaction, indoor air quality, work productivity

## Abstract

High indoor air quality is crucial for the health of human beings. The purpose of this work is to analyze the synergistic effect of particulate matter 2.5 (PM_2.5_) and carbon dioxide (CO_2_) concentration on occupant satisfaction and work productivity. This study carried out a real-scale experiments in a meeting room with exposures of up to one hour. Indoor environment parameters, including air temperature, relative humidity, illuminance, and noise level, were controlled at a reasonable level. Twenty-nine young participants were participated in the experiments. Four mental tasks were conducted to quantitatively evaluate the work productivity of occupants and a questionnaire was used to access participants’ satisfaction. The Spearman correlation analysis and two-way analysis of variance were applied. It was found that the overall performance declined by 1% for every 10 μg/m^3^ increase in PM_2.5_ concentration. Moreover, for every 10% increase in dissatisfaction with air quality, productivity performance decreased by 1.1% or more. It should be noted that a high CO_2_ concentration (800 ppm) has a stronger negative effect on occupant satisfaction towards air quality than PM_2.5_ concentration in a non-ventilated room. In order to obtain optimal occupant satisfaction and work productivity, low concentrations of PM_2.5_ (<50 μg/m^3^) and CO_2_ (<700 ppm) are recommended.

## 1. Introduction

People spend more than 80% of their time in enclosed buildings [1,2]. Hence, the indoor air quality has a great influence on occupants’ feelings, health, and work productivity. Existing studies suggest that investments in improvements of the indoor environment, especially the indoor air quality, could be financially feasible [3,4]. In order to improve the occupant satisfaction and work productivity, it is important to understand how the indoor environment affects them.

Most of existing studies focus on investigating the impact of temperature on building occupants. Wyon (1996) concluded that individual control of the air temperature helps to reduce the risk of sick building syndrome (SBS) and improve work productivity [5]. Toftum et al. (2002) showed that a decrease in air temperature from 23 °C to 18 °C provides better satisfaction towards air quality. In the meantime, it has no significant influence on the risk of sick building syndrome (SBS) for a short time [6]. Seppänen et al. (2006) found that work productivity at 30 °C is 91% of that at 22 °C [7]. Wong et al. (2008) proposed a series of logistic models, which are based on data from 293 occupants, to predict the acceptance of indoor environment quality in offices. In those models, operative temperature, CO_2_ concentration, the noise level, and the illumination level are included [8]. Experiments in Lan et al. (2011) indicated that a bit below thermal neutral leads to maximum performance and thermal discomfort causes lower performance [9]. In contrast, Wang et al. (2018) showed that the best performance is obtained when participants are under slightly warmer conditions [10]. Geng et al. (2017) established a quantitative relationship between work productivity and thermal environment to predict the relative work productivity according to the background air temperature. It shows that the highest thermal satisfaction is obtained at about 25 °C [11].

However, the air temperature is not the only influencing factor. The indoor air quality can also exert significant influences on the satisfaction and work productivity of occupants. The ventilation rate as well as the indoor and outdoor air pollutant levels are three dominant physical parameters in studies on the indoor air quality [12]. The main air pollutants include particulate matter 2.5 (PM_2.5_), carbon monoxide (CO), carbon dioxide (CO_2_), and the volatile organic compound (VOC). Among them, PM_2.5_ is “fine particles” with an aerodynamic diameter less than or equal to 2.5 microns (μm) [13].

In the past two decades, the economy has grown rapidly in China, while it has also been accompanied by serious air pollution. More and more attention is being given to the air quality, especially the PM_2.5_ level, since PM_2.5_ is one of the most important indexes to evaluate air quality. The ratio of the indoor to the outdoor PM_2.5_ concentrations (Indoor/Outdoor ratio) is a commonly used indicator to evaluate the indoor PM_2.5_ pollution [14,15,16,17].

Another indicator, CO_2_ concentration, is also often used to quantify the indoor air quality and ventilation [18]. The CO_2_ concentration in a room can be predicted based on the age and activity level of occupants. According to an IPCC (Intergovernmental Panel on Climate Change) report (2015), the concentration of CO_2_ in the atmospheric boundary layer is close to 400 ppm [19]. The American Society of Heating, Refrigerating and Air-Conditioning Engineers (ASHRAE) set 1000 ppm as a guideline value for CO_2_, based on a ventilation rate of 15 cfm/p and an outdoor CO_2_ concentration of 300 ppm [20]. It is worth mentioning that the CO_2_ level of 1000 ppm is a guideline for comfort acceptability rather than a mandatory requirement for indoor air quality [21].

Wargocki et al. (1999) suggested that indoor air pollution may reduce the performance of occupants [22]. Since then, the impact of indoor air quality on work performance has attracted the attention of many scholars. Wargocki (2000) confirmed that indoor air quality affects work productivity. Every 10% improvement of occupant dissatisfaction towards air quality helps to increase work productivity by 1.5% [23]. Based on a series of experiments, Wyon (2004) concluded that bad indoor air quality leads to a reduction of office performance of 6–9% [24]. Mui et al. (2009) reported that formaldehyde (HCHO) exposure risk increases by 2.5% for every 10-ppm increment, when the background CO_2_ concentration ranges from 800 to 1000 ppm [25]. Satish et al. (2012) found that more than half of subjects appear to reduce decision-making performance at CO_2_ concentrations of 1000 ppm, relative to 600 ppm [26]. Vehviläinen (2016) showed that higher CO_2_ concentrations reduce functional abilities [27]. Allen et al. (2016) found that cognitive performance is much better in green buildings than in conventional buildings and that high concentrations of VOCs and CO_2_ have a negative impact on work productivity [28].

Most of these previous studies only provide qualitative results, and hence it is difficult to provide a quantitative guideline on the control of indoor air quality. Also, the probable synergistic effect of PM_2.5_ and CO_2_ concentration still lacks investigations. Therefore, comprehensive studies considering the influences of air quality on occupant satisfaction and work productivity are badly needed.

The purposes of this study are:

- To evaluate the probable synergistic effect of CO_2_ and PM_2.5_ concentrations on occupant satisfaction towards air quality and work productivity.
- To provide guidance on how to improve occupant satisfaction and work productivity by controlling the indoor environment.

In order to achieve these purposes, therefore, a real-scale experiment with background conditions being carefully controlled was carried out. The collected data was tested by the Shapiro–Wilk method to determine the normal distribution. In addition, the relationship between the satisfaction and environmental parameters was considered and also the relationship between air quality dissatisfaction and performance changes. Besides, the results of the impact of PM_2.5_ and CO_2_ on occupants’ satisfaction and work productivity were demonstrated.

## 2. Methodology

This study focuses on the impact of the PM_2.5_ concentrations on the occupants. Both the PM_2.5_ and CO_2_ concentrations were involved dur to the synergistic effect produced by the CO_2_ concentration. The detailed experiment setup and procedures are described in this section.

### 2.1. Experiment Setup

**Figure 1:**
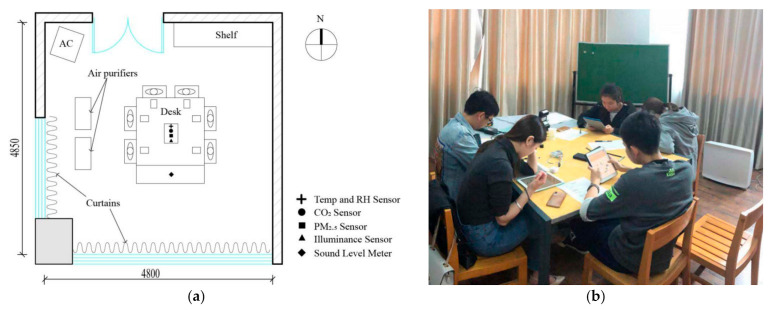
(**a**) Layout of the simulated meeting room; (**b**) Photo during the experiment.

A meeting room in an office building was used for carrying out the experiments. The layout of this room is 4.85 m by 4.80 m, which is shown in Figure 1.

The indoor environmental conditions were controlled as follows during the experiments. Windows and curtains were closed during the tests. The indoor temperature was controlled at around 24 °C to 26 °C by an air conditioner, with a relative humidity range of 40–60%. In this temperature and relative humidity set up, the occupants’ thermal dissatisfaction is at its lowest level [11]. Based on the standard for the lighting design of buildings [29], the illuminance on the desk was adjusted to 300 lux. In addition, the A-weighted sound pressure level was maintained at around 40 dB. The thermal, light, and acoustic conditions of the room were similar to common offices.

Before each experiment, when the outdoor PM_2.5_ reached the experimental target, the windows were opened to provide adequate natural ventilation to ensure the indoor PM_2.5_ and CO_2_ concentrations were similar to those of the outdoor environment. Then, the windows were closed and the indoor air circulation in the indoor environment was purified by two air purifiers (Air-O-Swiss P380 Plaston Group, Widnau, Switzerland). Five different levels of the intended PM_2.5_ concentrations (10, 25, 35, 50, 75 μg/m^3^) were selected. A series of preliminary experiments were conducted, showing that the PM_2.5_ concentration can be controlled within plus or minus 15% of the set value for up to one hour, when the indoor PM_2.5_ concentration is higher than 10 μg/m^3^. The limit values of PM_2.5_ concentrations were set with reference to WHO guidelines [30] and the air quality standards in China [31]. After the air was decontaminated by the particulate air purifiers, the measured PM_2.5_ concentration values typically reached the set point within one hour and remained at this value throughout the experiments. The indoor CO_2_ concentration level was gradually increased from approximately 500–900 ppm as the participants exhaled.

All relevant indoor environmental factors, as well as CO_2_ and PM_2.5_ factors, were monitored and recorded by the instruments at the center of the desk. Positions of those instruments are marked in Figure 1. Detailed information of instrument, including model types, and accuracies, are listed in Table 1. The accuracy of the PM_2.5_ nephelometer has been verified by comparing the measurement results from a DustTrak II aerosol monitor [TSI-8530, TSI Incorporated, Shoreview, MN, USA]. The calibrations were also performed at different temperatures and humidity, and different PM_2.5_ concentrations, and the results were found using the weighting method to adjust the measurement of Nephelometers. In addition, the particle count concentration monitoring was used, while three other offices were selected next door for validation. Two condensation particle counters (TSI CPC3007, TSI Incorporated, Shoreview, MN, USA) with a large concentration of particles larger than 0.01 μm in diameter and an accuracy of ±20% were used. The particle number concentration measurements were considered to be accurate at up to 100,000 cm^3^) were measured simultaneously and the results were similar. This calibration method has been recognized by other studies [32]. Besides, the outdoor air temperature, relative humidity, CO_2_ concentration, and PM_2.5_ was measured outside the building and the instruments were protected from direct sunlight, rainfall, pedestrians, traffic, and factors that would affect the accuracy of the data.

### 2.2. Participants and Experiment Procedure

Twenty-nine volunteers, 11 males and 18 females, were recruited in Zhejiang University. All participants are students of Zhejiang University. The Information on gender, age, height, weight, and body mass index (BMI) was collected, as summarized in Table 2. The BMI was calculated from weight and height [weight (kg)/height (m^2^)] [33]. For adults over 20 years old, normal BMI ranges from 18.5 to 24.9 kg/m^2^ [34]. The sample size of volunteers was referenced from previous studies in simulated environments, as shown in Table 3.

Five or six participants were randomly assigned to each of the five experimental groups. All participants were healthy and in a good mental state during the tests. They were all briefed on the experiment procedure before each test. During the test, they were allowed to adjust their clothes as they like and they had 15 min of adaptive time [10,11].

**Figure 2:**
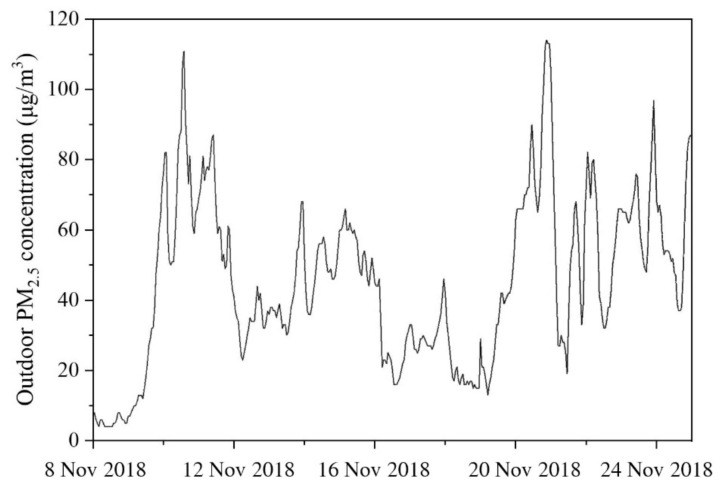
Outdoor PM_2.5_ concentration during experiments.

The experiments were carried out from 8 to 24 November 2018. The outdoor air temperature during the experiments was around 11–17 °C relative humidity was 40–90%, and CO_2_ concentration was 400–500 ppm, while the outdoor PM_2.5_ concentration during experiments is shown in Figure 2.

**Figure 3:**
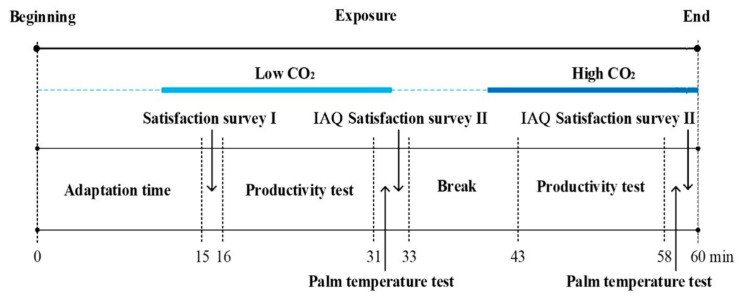
The procedure of each experiment. Note: The timeline is not consistent with the actual time.

Each experiment included three parts: a questionnaire survey, productivity test and palm temperature test, which is illustrated in Figure 3. Each productivity test consisted of four productivity tasks. Indoor environment parameters were recorded during the experiment. Palm temperature was used to determine the temperament of participants [36].

Each group of participants was subjected to five experimental conditions with five levels of PM_2.5_ concentrations (10, 25, 35, 50, 75 μg/m^3^). The sequence of the experimental conditions was determined. In order to eliminate the potential influences of group number and scenario order, the Latin-square design was used, as shown in Table 4. The procedure of each experiment (m = 1,2,…,5) included four stages: adaptation, a productivity test with low CO_2_ concentration(S_mL_) conditions, a break, and a productivity test with high CO_2_ concentration(S_mH_) conditions. The adaption time was set to 15 min after the participants entered the testing room. As there was a negligible difference between the indoor and the outdoor environment, both of the productivity tests lasted for 15 min and with a 10 min break. At the end of the adaptation, a satisfaction questionnaire survey of the indoor environment was carried out to rule out discomfort regarding the environmental factors. After each productivity test, the palm temperature of each participant was recorded by an infrared thermometer (MT4 max, Fluke corporation, Everett, WA, USA) [36], and the IAQ (Indoor Air Quality) satisfaction questionnaire survey was conducted. The experiments followed a single-blind process, where the experimental conditions were unknown to the participants.

The satisfaction questionnaire survey (Appendix A) consists of Environmental satisfaction and IAQ satisfaction. The participants’ satisfaction has different factors, including indoor and outdoor air quality, temperature, relative humidity, lighting, acoustics, and overall environment. Satisfaction was rated on a 7-point scale ranging from “very satisfied (+3)” to “very dissatisfied (−3)”, with a neutral midpoint (0) [37]. Votes of −3 to −1 were classified as air quality dissatisfaction, and the percentages of dissatisfaction were calculated separately. The IAQ Questionnaires survey was recorded twice, considering the low and high CO_2_ concentrations.

The productivity test consisted of four tasks: “Recognition of Figures”, “Stroop Color and Word”, “Rule-based Reasoning”, and “Schulte Grid 7 × 7” (Table 5). They are used to measure different aspects of work productivity, including understanding and memory, perception, logical thinking, and visual attention [38].

Recognition of Figures is used to measure the ability of understanding and memory [39]. Twenty-four pages with up to 10 patterns appear in a sequential order. Participants need to find a pattern that has never appeared before. Only one chance is given in each page. Up to three mistakes are allowed in each task. Participants get 200 points for each page passed, and the scores are calculated automatically.

● The Stroop Color and Word is a neuropsychological test used to assess the perception ability [40]. Two words are displayed on the screen at the same time. The words name a color that is not the same as the ink color of the word; for example, the word “blue” is displayed in red ink. Participants need to determine if the color described by the first word is the same as the ink color in which the second word is displayed. Participants have 45 s in each task. They get 50 points for each correct answer, and a 50 points penalty for each wrong answer.
● Rule-based Reasoning is used to evaluate logical thinking ability [41]. There are five groups of geometric patterns in different colors. Each group has 10 patterns, which have a common color or shape characteristic. Participants need to determine whether each pattern conforms to a certain rule through trial and error. Participants get 50 points for each correct answer, and no penalty for a wrong answer.
● The Schulte Grid was developed originally as a psycho-diagnostic test to study the properties of attention by German psychiatrist and psychotherapist Walter Schulte [42]. It was used to evaluate the visual attention in this study [43]. At the beginning, the screen displayed a 7 × 7 grid table with 49 randomly distributed numbers. Participants touched a sequential series of numbers in ascending values as quickly as possible. At the end of task, the actual finish time was calculated and recorded automatically. The reciprocal of finish time was used to represent the performance of visual attention.

The detailed tested parameters for four tasks are summarized in Table 5. All participants performed four tasks on an iPad. After the test, the scores and finish times were automatically calculated and recorded by the predetermined program. To encourage all participants to try their best, the higher score was associated with a better bonus.

### 2.3. Statistical Analysis Methods

The recorded data of the satisfaction of the participants regarding the indoor environment and performance data in the productivity test were both analyzed with SPSS [SPSS.20, IBM Corporation, New York, NY, USA]. The Shapiro–Wilk test was first used to check whether the satisfaction and performance data were normally distributed [44]. The Spearman correlation analyses between the satisfaction vote and indoor environment parameters were carried out to evaluate the degree of correlation. Afterwards, a two-way analysis of variance (two-way ANOVA) was adopted to compare the satisfaction vote and performance of productivity results under each experiment conditions. The significance level of these tests was set as 0.05. The results are statistically significant if the *p*-value (*p*) is less than 0.05. The effect size (*ES*) was calculated to explain the sizes of differences between each group. It indicates whether the difference is practically important [45]. In this study, partial eta squared, denoted as partial η^2^, was used to represent the *ES*. Partial η^2^ is a proportion of variance accounted for by some effect. Partial η^2^ of 0.01, 0.06, and 0.14 for two-way ANOVA indicate the small, moderate, and large effect sizes (*ES*) [46].

The palm temperature of the participants was expected to be stable, which indicates that their mental state is stable. The palm temperatures are standardized with Equation (1) to compare the average value of each subject in different conditions:

(1) $$T_{i,j}^{\prime }=T_{i,j}\times 100\%/\left(\overset{n}{\underset{j=1}{\sum }}T_{i,j}/n\right)$$

where $T_{i,j}$ is the palm temperature of participant *i* in experimental condition *j*, *n* is the number of experimental conditions for each subject (*j* = 2 m^−1^ for low CO_2_ concentration, *j* = 2 m for high CO_2_ concentration), and $T_{i,j}^{\prime }$ is the standardized value of participants’ palm temperature *i* in the experimental condition *j*. Based on this premise, the following indicators were applied to illustrate the satisfaction and productivity levels of participants at different PM_2.5_ concentrations.

- Dissatisfaction rate (*R*_dis_) and mean satisfaction vote $\bar{SV}$.

The votes record for both environment and IAQ were classified as dissatisfaction when the vote record was very dissatisfied, dissatisfied, or slightly dissatisfied. The dissatisfaction rate as a percentage of all votes cast is the dissatisfaction rate (*R*_dis_).

The votes ranging from very dissatisfied to very satisfied were assigned from −3 to +3 with the neutral point 0. The mean vale of the satisfaction votes ($\bar{SV}$) indicated the satisfaction degree of the occupants in each experimental condition.

- Standardized score and relative performance

The scores of each productivity task were standardized with Equation (2) to compare the average value of each subject in different conditions:

(2) $$z_{i,j}^{\prime }=n\times z_{i,j}\times 100\%/\left(\overset{n}{\underset{j=1}{\sum }}z_{i,j}\right)$$

where $z_{i,j}$ is the scores or the reciprocals of finish times for participant *i* in experimental condition *j*, *n* is the number of experimental conditions for each participant, and $z_{i,j}^{\prime }$ is the standardized value of participant *i* in experimental condition *j*.

The relative performance (*RP*) is the average value of standardized scores of the four tasks, which was used to evaluate the overall work productivity:

(3) $$RP_{i,j}=\sum_{k=1}^{4}z_{i,j,k}^{\prime }\times 100\%/4$$

where $z_{i,j,k}^{\prime }$ is the standardized performance of subject *i* in experimental condition *j*, *k* is the number of tasks, and $RP_{i,j}$ is the relative performance of participant *i* in experimental condition *j*.

## 3. Influence of PM_2.5_ and CO_2_ on Occupants’ Satisfaction

### 3.1. Measured Indoor Environment Parameters

Under the conditions of natural ventilation, the indoor CO_2_ concentration ranges from 500 ppm to 600 ppm. Also, the indoor PM_2.5_ concentration is the same as the outdoor PM_2.5_ concentration. Table 6 shows the measured indoor environment parameters of five design scenarios. The PM_2.5_ concentration in each test was controlled within plus or minus 15% of the designed value. Curtains were always closed to block direct sunlight. The air temperatures of five design scenarios were kept at about 25 °C. CO_2_ concentration started at about 600 ppm and ended at 800–900 ppm in each experiment. The illuminance on the desk was controlled at about 300 lux. The A-weighting sound pressure was 38–43 dB.

### 3.2. Satisfaction Votes with Different PM_2.5_ Concentrations

**Figure 4:**
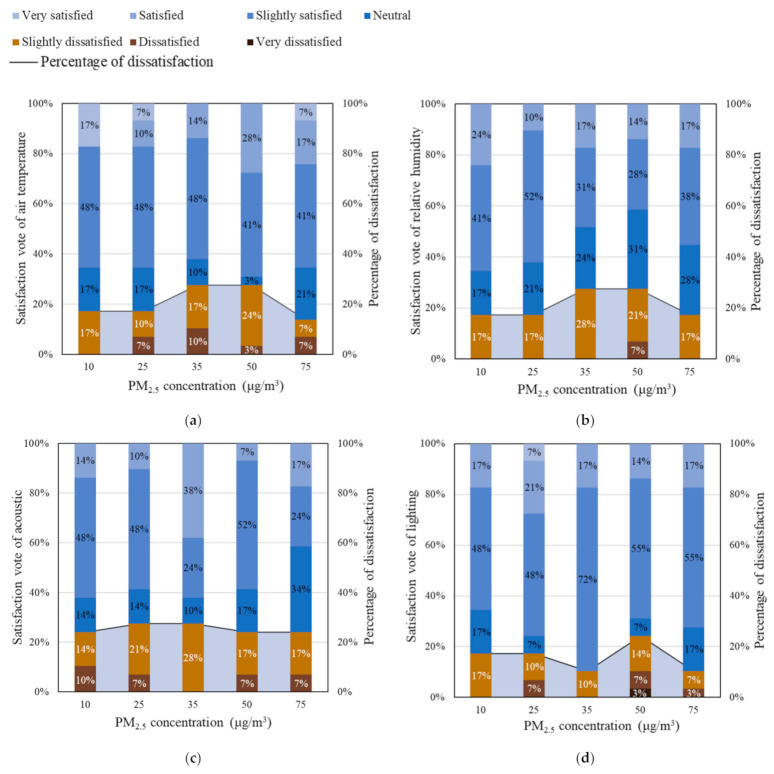
Distribution of Satisfaction vote and percentage of dissatisfaction of indoor environmental factors.

According to Equation (1), the standardized value of palm temperature was calculated, as shown in Table 7. It can be found that there is no significant difference in palm temperature under different PM_2.5_ and CO_2_ concentrations (*p* > 0.05). The standard deviations are all equal or below 3%. The mental states of participants were generally consistent through the experiment. The satisfaction results in Figure 4 suggest that there is no significant correlation between PM_2.5_ concentration and the satisfaction vote of air temperature, relative humidity, acoustics, and lighting environment. Less than 30% were dissatisfied with the air temperature, relative humidity, lighting, and acoustic environment. This means that most participants were satisfied with the indoor air temperature, relative humidity, lighting, and acoustics.

**Figure 5:**
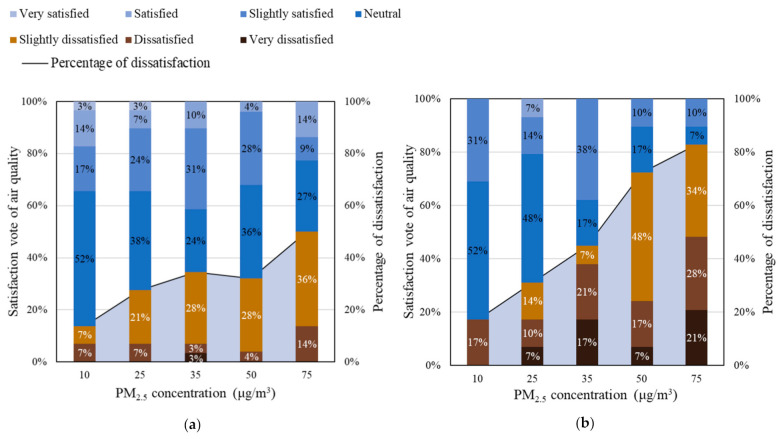
Distribution of satisfaction vote and percentage of dissatisfaction on air quality under different CO_2_ concentration:

The satisfaction votes results for indoor air quality under different PM_2.5_ concentrations are illustrated in Figure 5. The percentage of dissatisfaction can be evidently correlated to the PM_2.5_ concentration. At the lowest PM_2.5_ concentration (10 μg/m^3^), the IAQ dissatisfied rate (*R*_dis_) was only 14% and 17% for the low and the high CO_2_ concentrations, respectively. It increased gradually with the increase in the PM_2.5_ concentration, reaching up to 50% and 83% for low CO_2_ and high CO_2_, when the PM_2.5_ was at 75 μg/m^3^. Along with the PM_2.5_ concentration, the high CO_2_ concentrations also contributed to the increase in the dissatisfied rate. When the CO_2_ concentrations were within the range of 550–700 ppm, each 1 μg/m^3^ increment in the PM_2.5_ concentration increased the IAQ dissatisfied rate by 0.5%. The CO_2_ concentrations were within the range of 750–950 ppm, while the IAQ dissatisfied rate increased by 1.1% per unit of the PM_2.5_ concentration. Based on the Spearman correlation analysis, the correlation coefficient between satisfaction vote of air quality and PM_2.5_ concentration was −0.26 (*p* < 0.05), and that between the satisfaction vote of air quality and CO_2_ concentration was −0.29 (*p* < 0.05). It can be concluded that satisfaction vote of air quality is affected by both PM_2.5_ and CO_2_ concentrations.

**Figure 6:**
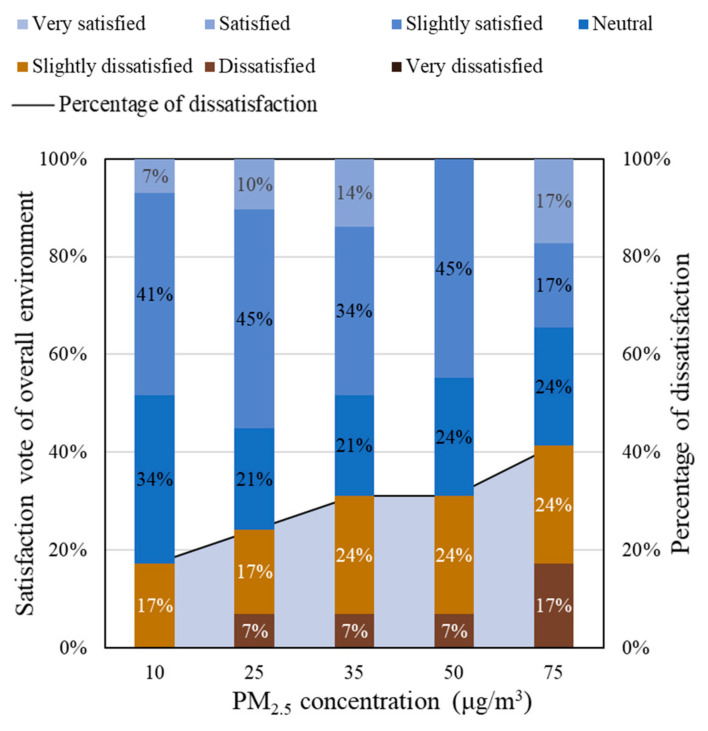
Satisfaction vote and percentage of dissatisfaction of overall environment.

The dissatisfaction caused by high PM_2.5_ concentrations also contributes to the decline in occupants’ satisfaction with the overall indoor environment (Figure 6). When the PM_2.5_ concentration rose from 10 to 75 μg/m^3^, the overall indoor environmental dissatisfaction rate increased from 17% to 41%. According to Figure 5, each 1% increment in the indoor air quality dissatisfaction would result in a 0.5% rise in the overall environment dissatisfied rate. The PM_2.5_ concentration has a remarkable impact on satisfaction vote towards the overall environment.

### 3.3. Satisfaction Vote of Air Quality under Different PM_2.5_ Concentrations

**Figure 7:**
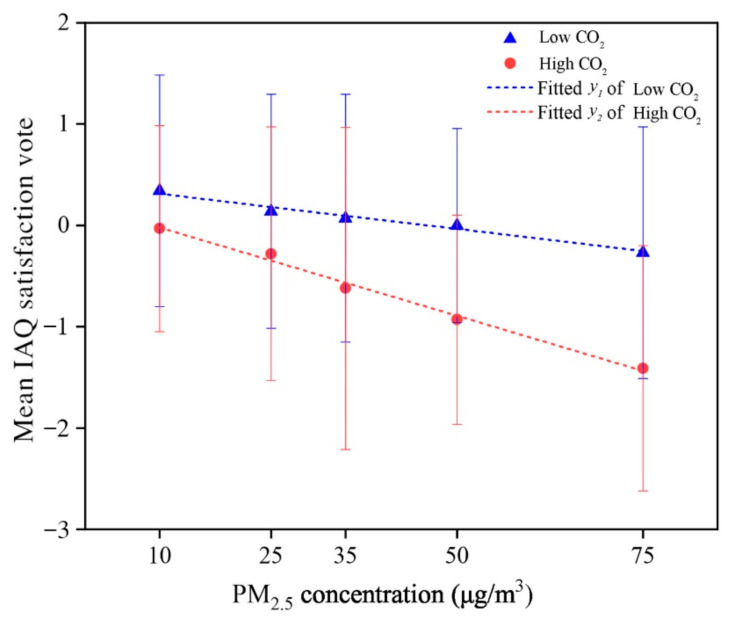
The relationship between mean IAQ satisfaction vote and PM_2.5_ concentration.

The mean values of the IAQ satisfaction votes ($\bar{SV}$) under the different PM_2.5_ concentrations for the votes for satisfaction are shown in Figure 7. Under the low CO_2_ concentration condition, IAQ satisfaction votes decreased from 0.34 to −0.27 when the PM_2.5_ concentration rise from 10 to 75 μg/m^3^. Under the high CO_2_ concentration condition, the IAQ satisfaction votes decreased from −0.03 to −1.41 within the range of the PM_2.5_ concentration from 10 to 75 μg/m^3^. The results of changes in IAQ satisfaction votes consistent with the results shown in Figure 5, that showing the significant effect of indoor PM_2.5_ concentration on occupant satisfaction. The effect is simultaneously exacerbated when combined with a high CO_2_ concentration.

The fitting lines of predicted mean IAQ satisfaction vote and PM_2.5_ concentration under two CO_2_ scenarios are expressed as follows:

For those cases with a low CO_2_ concentration (450–700 ppm):

(4) $${\bar{SV}}_{IAQ}=-0.0087C_{PM2.5}+0.40\;\mathrm{for}\;10\;\leq \;C_{PM2.5}\;\leq 75\;\mu g/m^{3},\;R^{2}=0.97$$

For those cases with a high CO_2_ concentration (720–900 ppm):

(5) $${\bar{SV}}_{IAQ}=-0.022C_{PM2.5}+0.20\;\mathrm{for}\;10\;\leq \;C_{PM2.5}\;\leq 75\;\mu g/m^{3},\;R^{2}=0.99$$

where *C_PM_*_2.5_ is the PM_2.5_ concentration and ${\bar{SV}}_{IAQ}\;$ is the predicted mean value of the IAQ satisfaction vote.

According to Equations (4) and (5), it was found that the mean IAQ satisfaction vote declines faster at a high CO_2_ concentration level (720–900 ppm) than at a low CO_2_ concentration level (450–700 ppm). In other words, the increase rate of dissatisfaction with PM_2.5_ is exaggerated by the higher CO_2_ concentration. More specifically, on condition of a low CO_2_ concentration (450–700 ppm), if the PM_2.5_ concentration is less than 50 μg/m^3^, the mean IAQ satisfaction vote is above zero; on the condition of a high CO_2_ concentration (720–900 ppm), the average satisfaction vote of the air quality is consistently below zero. Therefore, in order to achieve an IAQ satisfaction vote above zero, it is recommended that PM_2.5_ concentrations be maintained at 50 μg/m^3^ or less, while CO_2_ concentrations can be limited to 700 ppm or less.

## 4. Influence of PM_2.5_ and CO_2_ on Work Productivity

### 4.1. Work Productivity with Different PM_2.5_ and CO_2_ Concentration

The standardized performances of four tasks, obtained with Equation (2), are summarized in Table 8. According to the Shapiro–Wilk test, except for logical thinking when PM_2.5_ concentration was 10 μg/m^3^, all the data were normally distributed. The CO_2_ concentration influence was excluded from the two-way analysis of variance (ANOVA), leaving only the impact of the PM_2.5_ concentration, where the df is degree of freedom, *F* is the variance analysis results, *ES* is the effect size, and the *p*-value is the mean square.

**Figure 8:**
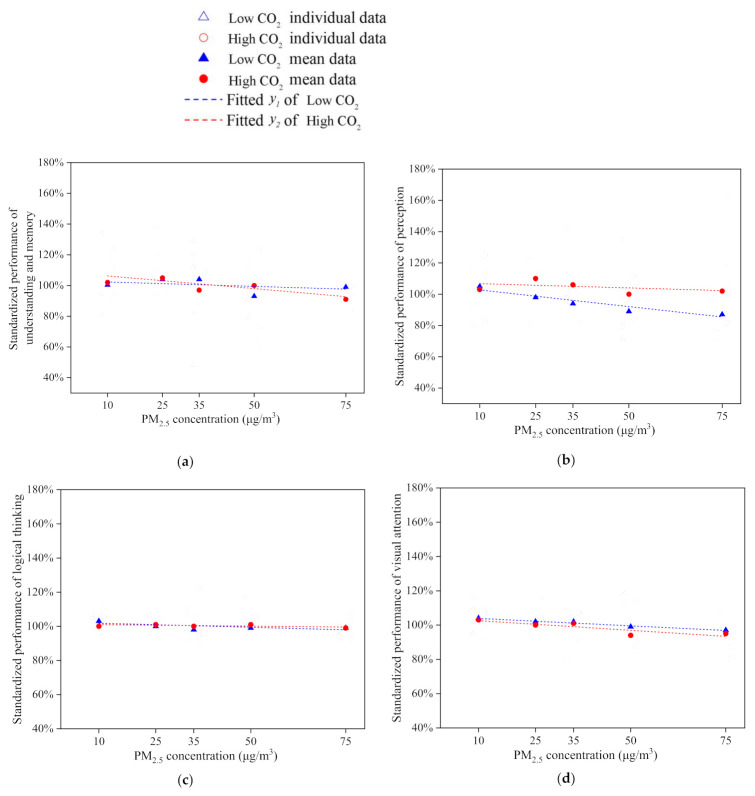
Standardized performances of four productivity tests.

Figure 8 shows that the trend of each task is different. According to Equation (2), 100% is the average level of each subject in productivity tests. There is no significant difference in understanding and memory or logical thinking. The standard deviations in logical thinking are less than 10%. These are much lower than for other tasks. A high CO_2_ concentration of 800 ppm can reduce occupant satisfaction (Figure 6), but no obvious evidence shows that it would influence the performance of understanding and memory or logical thinking, compared with that under the low CO_2_ concentration.

The *p*-value and the effect size were applied to identify the significance level and the difference between the different experimental conditions. The p-values for the task of perception were less than 0.05. *ES*s for perception were less than 0.06 but larger than 0.01. That explains why PM_2.5_ concentrations have a significant effect on the performance of understanding and memory, perception, and visual attention, as *p* < 0.05. The effect sizes are considered to be important as *ES* > 0.01. Combined with Figure 8, the performances of understanding, perception, and visual attention decreased as the PM_2.5_ concentration increased with the same level of CO_2_ concentration.

Especially in the visual attention task, the *ES* of PM_2.5_ concentration on visual attention is more than 0.06. Therefore, there is a moderate effect on visual attention. The performance standardized scores decreased from 104% to 96%, while the PM_2.5_ concentration increased from 10 to 75 μg/m^3^. This indicates that a high CO_2_ concentration reduces the visual attention. On the logical thinking task, the *p*-value is larger than 0.05, which means the decrease was not significant, but the effect size presented some practical importance for values greater than 0.01. The impact of the indoor PM_2.5_ concentration on mental work was thus verified.

### 4.2. Relative Performance under Different PM_2.5_ Concentrations

Many air quality experiments have been conducted to evaluate the impact of air pollution on performance change. Zivin and Neidell (2012) concluded that the impact of ozone on productivity is significant. A 10-ppb increase in ozone exposure directly leads to a 5.5% decrease of agricultural outcomes [47]. Adhvaryu et al. (2014) reported that an increase of 10 μg/m^3^ in air pollution leads to a reduction of 0.3% in worker efficiency in an Indian factory [48]. Chang et al. (2016) reported that productivity among fruit workers drops by 6% with a 10-unit increase in PM_2.5_ in California [49]. Another report from Chang showed that a 10-unit increase in the air pollution index (API) lowers the worker daily calls by 0.35% in China [50]. He et al. (2019) found that the effect of air pollution on labor productivity is subtle, and there is a 95% probability that every 10 μg/m^3^ in PM_2.5_ concentration causes a performance change from −0.4% to 0.1% [51].

**Figure 9:**
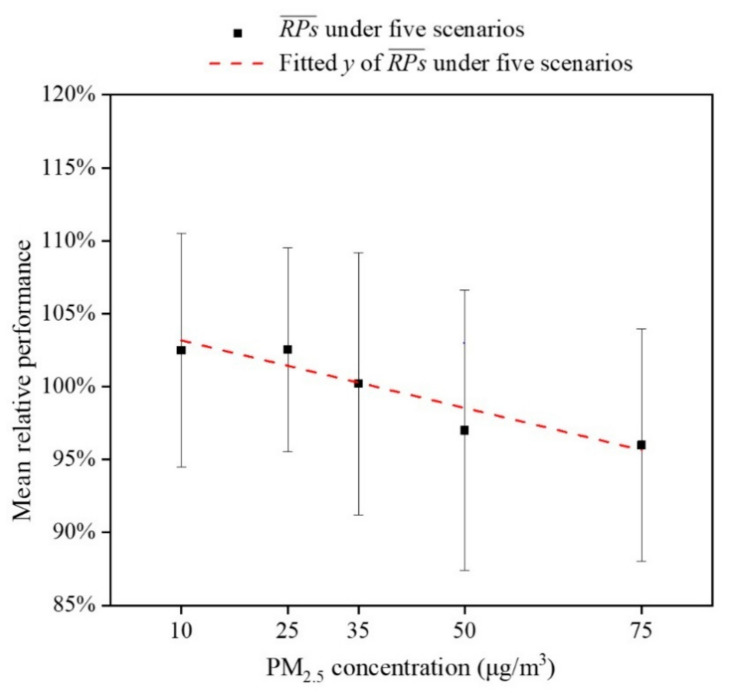
Comparison of the quantitative relationship between relative performance and PM_2.5_ concentration.

The productivity of the participants under experimental condition was weighted with the relative performance (*RP*_i,j_) in Equation (3). The relationship between mean value of *RP* with different PM_2.5_ concentration is shown in Figure 9. In general, with an increase of PM_2.5_ concentration, the *RP* slightly decreases from 103% to 96%. The predicted mean value of the relative performance can be calculated by the least square method and the fitting relationship is as follows:

(6) $$\bar{RP}=-0.001C_{PM2.5}+1.04\;\mathrm{for}\;10\;\leq \;C_{PM2.5}\;\leq \;75\;\mu g/m^{3},\;R^{2}\;=\;0.897.$$

For comparison, the ratio of the change in RP to the change in PM_2.5_ concentration is defined to estimate the effect of the PM_2.5_ concentrations on productivity, expressed as Equation (7):

(7) $$\alpha =\Delta \bar{RP}/\Delta C_{PM2.5}$$

where $\Delta \bar{RP}\;$ is the changes in the predicted mean value of the relative performance; $\Delta C_{PM2.5}\;$ is the changes in PM_2.5_ concentrations; α is the ratio of the decrease in the productivity to the increase in the PM_2.5_ concentration.

**Figure 10:**
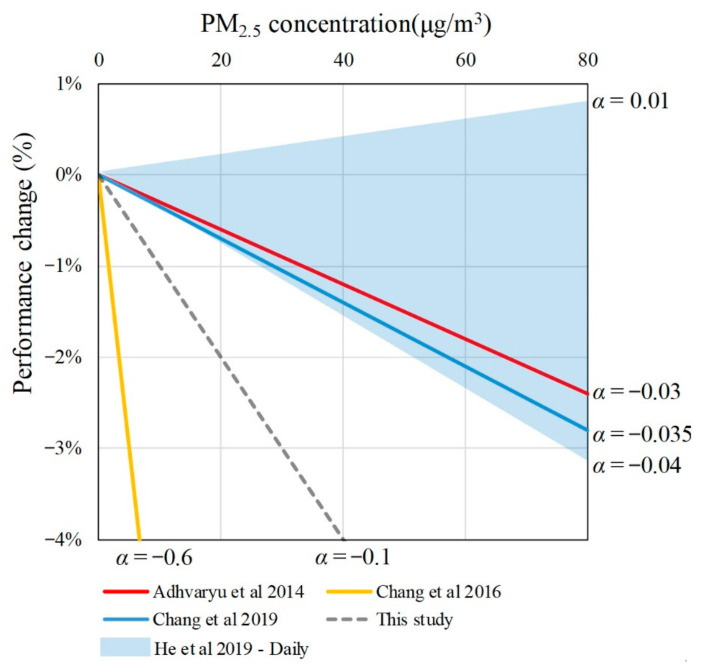
Comparison of the relationship between PM_2.5_ concentration and performance change. Data sources: Adhvaryu et al. 2014 [48]; Chang et al. 2019 [50]; This study; Chang et al. 2016 [49]; He et al. 2019 [51].

The smaller the α value (the greater the absolute value of a negative number), the greater the rate of decrease in the work productivity. According to previous experiments (Table 9 and Figure 10), Adhvaryu et al. [48] discussed the impact of the indoor PM_2.5_ concentration on the productivity in a garment factory, where the α value was −0.03%. Other two studies carried out in a Chinese call center and manufacturing firms shows the α value was −0.035% and [−0.04 to 0.01%]. The α value in this study was −0.10%, which was lower than these studies. Compared with these studies, this indicates that the mental work in offices tends to be more sensitive to PM_2.5_ pollution, while low-intensity workers tend to be more sensitive to PM_2.5_ pollution than high-intensity workers. Based on a study in a pear-packing factory in United States (α value = −0.6), it seems that workers in China and India have a higher tolerance towards PM_2.5_ pollution. This may be related to the environment that the workers have been in for a long time, where smaller changes in air quality in a better environment may cause a decrease in the productivity. Conversely, changes in air quality have relatively little impact on productivity as workers adapt to already poor environment.

### 4.3. Relationship between Air Quality Dissatisfaction and Performance Change

**Figure 11:**
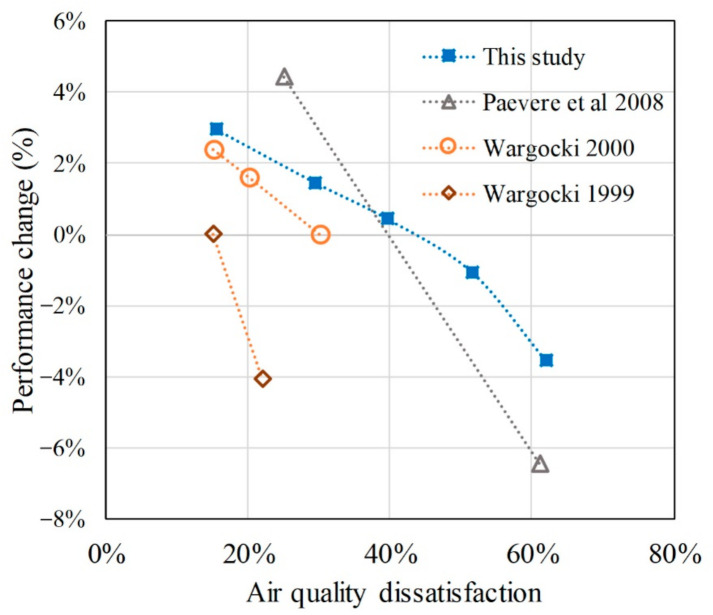
Comparison of the relationship between air quality dissatisfaction and performance change. Data sources: [22]; Wargocki 2000 [23]; Paevere et al. 2008 [50].

The relationship between the productivity of the participants and IAQ satisfaction under the different PM_2.5_ conditions was further analyzed. The Figure 11 shows the air quality dissatisfaction and performance change between this study and previous work. In this study, every 10% increase in the IAQ dissatisfaction would reduce the work productivity by 1.3% when the IAQ dissatisfaction value is lower than 40%. However, when the IAQ dissatisfaction increases over 40%, the downward trend of the work productivity is more pronounced. Every 10% increase in the IAQ dissatisfaction would reduce the work productivity by 1.5%, and this number would increase to 2.6% when the IAQ dissatisfaction value rises over 50%. This result was consistent with the trends observed in the previous works [23,52].

**Figure 12:**
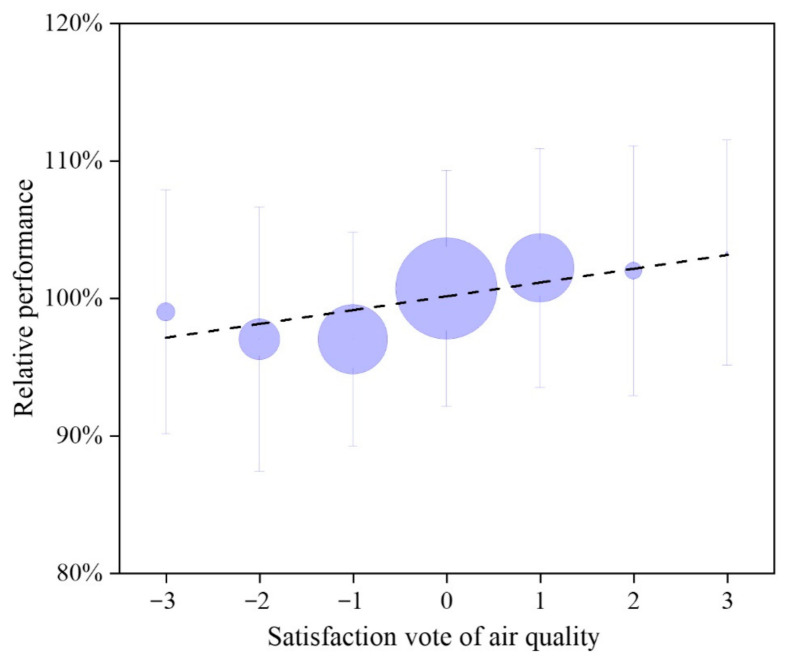
The quantitative relationship between relative performance and satisfaction vote of air quality. The size of circle represents the corresponding sample size.

The relative performance of the participants with the same number of satisfied votes is shown in Figure 12. The size of each circle represents the corresponding number of participants who voted for that degree of satisfaction. Most of air quality satisfaction votes ranged between −2 and 1, which means votes were between dissatisfied and slightly satisfied. A positive correlation can be observed between the mean value of RP and the satisfaction vote, while the fitting line expressed as Equation (8).

(8) $$\bar{RP}=-0.010SV_{IAQ}+1.00\;\mathrm{for}\;-3\;\leq \;SV_{IAQ}\;\leq 3,\;R^{2}=0.72$$

where $\bar{\;RP}$ is the mean value of the relative performance; $SV_{IAQ}\;$ is the participant IAQ satisfaction vote.

When the satisfaction vote is equal to or greater than zero, the predicted $\bar{\;RP}$ is more than 100%. Conversely, if the satisfaction vote is less than zero, the predicted $\bar{\;RP}$ is less than 100%. Considering the predicted mean IAQ satisfaction expressed in Equations (4) and (5), the indoor PM_2.5_ must be kept within 50 μg/m^3^ and the CO_2_ concentrations preferably limited to 700 ppm to improve the work productivity and the satisfaction in offices.

## 5. Conclusions

This study presents data from PM_2.5_ and CO_2_ exposure evaluations on occupant satisfaction and work productivity in a simulated meeting room. A quantitative model to assess work productivity was established, including focusing on understanding and memory, perception, logical thinking, and visual attention. The main conclusions are as follows:

1. The results indicate that every 1 μg/m^3^ increment of indoor PM_2.5_ concentration (in the range of 10–75 μg/m^3^) would increase the dissatisfied rate by 0.5% at a low CO_2_ condition and 1.1% at a high CO_2_ condition. This impact is exacerbated when coupled with a high CO_2_ concentration, as every 1% increase in the air quality dissatisfaction would causes a 0.5% increase in the overall environment dissatisfaction.
2. The impact of the high PM_2.5_ with CO_2_ concentrations on the participants performances in the four mental tasks was verified by statistical analysis. Every 10 μg/m^3^ increase in the PM_2.5_ concentration level can reduce the overall performance by 1%. The mental work tended to be more sensitive when compared with manual work.
3. It is suggested to maintain the indoor PM_2.5_ within 50 and CO_2_ concentration at less than 700 ppm in order to improve the work productivity and occupant satisfaction for indoor air quality in offices.

The participants in this study were all college students, and the impact on people of different ages and physical conditions may be different. Because this relationship may vary based on the ventilation mode, room layouts, and participants, further experiments are needed.

## Figures and Tables

**Table 1:** Measuring parameters and instruments.

Parameter	Instrument Model	Manufacturer	Measurement Principle	Accuracy
Air temperature and relative humidity (RH)	Self-recording hygro-thermometer	WSZY-1, Beijing Tianjianhua instrument technology development Co. Ltd., Beijing, China	Electronic induction	Temperature: ±0.2 °C RH: ±2%
CO_2_ concentration	CO_2_ sensor	Telaire 7001, Onset Computer Corporation, Bourne, MA, USA	Dual wavelength infrared	±50 ppm
A-weighting sound pressure level	Sound level meter	Aihua AWA6228+, Hangzhou Aihua Instruments Co., Ltd., Hangzhou, China	Frequency weighting, time weighting and pulses	±1.5 dB
Illuminance	HOBO data logger	U12-012, Onset Computer Corporation, Bourne, MA, USA	Photocells and ammeters	±4%
PM_2.5_ concentration	Nephelometers	QD-W1, Beijing Green Built Environment Technology Co., Ltd., Beijing, China	Laser light scattering	±5%

**Table 2:** Background characteristics for the 29 participants.

Gender	*N*	Age (y)	Height (m)	Weight (kg)	BMI (kg/m^2^)
Male	11	23.45 (1.13)	1.75 (0.04)	67.27 (6.08)	22.18 (2.52)
Female	18	23.67 (1.97)	1.63 (0.06)	52.94 (6.34)	19.94 (1.89)

Notes: N is sample size; means (standard deviations) are listed.

**Table 3:** Comparison of sample sizes in different studies.

Data Sources	Sample Size	Gender
Wargocki (1999) [22]	30	30 Females
Lan et al. (2011) [9]	12	6 Males and 6 Females
Liu et al. (2014) [35]	20	20 Males
Allen et al. (2016) [28]	24	10 Males and 14 Females
Geng et al. (2017) [11]	21	12 Males and 9 Females
Wang et al. (2018) [10]	12	6 Males and 6 Females
This study	29	11 Males and 18 Females

**Table 4:** Latin-square design for the experiment.

	S_1L_/S_1H_	S_2L_/S_2H_	S_3L_/S_3H_	S_4L_/S_4H_	S_5L_/S_5H_
Group 1	10 μg/m^3^	25 μg/m^3^	35 μg/m^3^	50 μg/m^3^	75 μg/m^3^
Group 2	25 μg/m^3^	35 μg/m^3^	50 μg/m^3^	75 μg/m^3^	10 μg/m^3^
Group 3	35 μg/m^3^	50 μg/m^3^	75 μg/m^3^	10 μg/m^3^	25 μg/m^3^
Group 4	50 μg/m^3^	75 μg/m^3^	10 μg/m^3^	25 μg/m^3^	35 μg/m^3^
Group 5	75 μg/m^3^	10 μg/m^3^	25 μg/m^3^	35 μg/m^3^	50 μg/m^3^

**Table 5:** Detailed recorded parameters for four tasks.

Task	Test Objective	Ending Condition	Record Parameters
Recognition of figures	Understanding and memory	Three mistakes	Scores
Stroop color and word test	Perception	45 s	Scores
Rule-based reasoning	Logical thinking	50 chances	Scores
Schulte Grid test 7 × 7	Visual attention	Touch from 1 to 49	Finish times

**Table 6:** Measured indoor environment parameter at the center of the desk.

Scenario (μg/m^3^)	PM_2.5_ (μg/m^3^)	Temperature (°C)	RH (%)	Illuminance (lux)	Acoustic (dB)	Separate Measured	
						Low CO_2_ (ppm)	High CO_2_ (ppm)
10	10.6 (1.0)	24.9 (0.5)	44.6 (4.6.)	309 (23.5)	40.7 (3.7)	630 (86)	863 (123)
25	25.2 (1.4)	25.1 (0.6)	41.8 (5.2)	328 (34.5)	42.8 (3.7)	595 (38)	794 (58)
35	34.7 (1.8)	25.2 (0.5)	40.8 (3.4)	296 (25.0)	42.3 (5.3)	618 (40)	857 (98)
50	50.3 (1.6)	24.9 (0.5)	42.3 (3.3)	323 (26.3)	38.6 (3.1)	608 (102)	850 (41)
75	73.1 (2.2)	24.8 (0.7)	46.6 (6.1)	329 (24.0)	41.6 (3.3)	653 (76)	899 (56)

Notes: Means (standard deviations) are listed.

**Table 7:** Standardized results of palm temperature; effect sizes (*ES*) of 0.01, 0.06, and 0.14 indicate small, moderate, and large effects.

	CO_2_ (ppm)	PM_2.5_ (μg/m^3^)					PM_2.5_		CO_2_	
		10	25	35	50	75	P	*ES*	P	*ES*
Palm temperature	Low	101% (2%)	100% (3%)	100% (3%)	100% (3%)	100% (2%)	0.22	0.01	0.83	0.01
	High	100% (2%)	100% (2%)	100% (3%)	100% (2%)	100% (2%)				

Note: means (standard deviations) are listed.

**Table 8:** Two-way analysis of variance (ANOVA) of four tasks performance (effect sizes of 0.01, 0.06, and 0.14 indicate small, moderate, and large effects).

Task	CO_2_ (ppm)	PM_2.5_ (μg/m^3^)					df	Mean Square	*F*	*p*	*ES*
		10	25	35	50	75					
Understanding and memory	Low	100% (15%)	104% (15%)	104% (20%)	93% (20%)	99% (19%)	4	0.092	3.582	0.007 *	0.052 ^#^
	High	102% (19%)	105% (10%)	97% (22%)	100% (17%)	91% (18%)					
Perception	Low	105% (18%)	98% (16%)	94% (22%)	89% (23%)	87% (16%)	4	0.134	3.071	0.017 *	0.045 ^#^
	High	103% (21%)	110% (19%)	106% (22%)	100% (29%)	102% (23%)					
Logical thinking	Low	103% (4%)	100% (5%)	98% (6%)	99% (6%)	99% (5%)	4	0.007	2.326	0.057	0.034 ^#^
	High	100% (7%)	101% (5%)	100% (8%)	101% (5%)	99% (5%)					
Visual attention	Low	104% (9%)	102% (10%)	102% (13%)	99% (9%)	97% (8%)	4	0.063	6.833	0.000 *	0.095 ^##^
	High	103% (10%)	100% (13%)	101% (10%)	94% (9%)	95% (10%)					

Note: * significant influence as *p* < 0.05. ^#^ small effect sizes (*ES* > 0.01) and ^##^ moderate effect sizes (*ES* > 0.06).

**Table 9:** Comparison of the rates of performance change in different studies.

Data Sources	Location	Environment	PM_2.5_ (μg/m^3^)	α (%)
Adhvaryu et al. (2014) [48]	India	Garment factory	(21,110)	−0.03
Chang et al. (2016) [49]	United States	Pear-packing factory	(1,21)	−0.60
Chang et al. (2019) [50]	China	Call center	(10,200) *	−0.035
He et al. (2019) [51]	China	Manufacturing firms	(3,237)	(−0.04,0.01)
This study	China	A meeting room	(10,75)	−0.10

Note. * The air pollution index was used in research of Chang et al. (2019) [50]. According to analysis from He et al. (2019) [51], it is assumed that 10 API points are approximately equal to 10 μg/m^3^.

## Data Availability

The data presented in this study are available on request from the corresponding authors. The data are not publicly available due to participants’ privacy.

## Nomenclature

*C*_PM2.5_	Concentration of PM_2.5_ (μg/m^3^)
*ES*	Effect size
*RP*	Relative performance
*SV*_AQ_	Satisfaction vote of air quality
*z*	The scores and reciprocals of finish times
*z′*	The standardized value of scores and reciprocals of finish times
*α*	Rate of performance change
*β*	The constants in different cases

## Appendix A

NO: _――――――_ TIME: _――――――_

           **Questionnaire Survey**

1. **Background information**
  (a) Name: _――――――_
  (b) Gender: □ Male □ Female
  (c) Age: _――――――_; Height: _――――――_cm; Weight: _――――――_kg
2. **Satisfaction survey I**

	Very dissatisfied Neutral Very satisfied						
	−3	−2	−1	0	1	2	3
Indoor air quality	○	○	○	○	○	○	○
Outdoor air quality	○	○	○	○	○	○	○
Air temperature	○	○	○	○	○	○	○
Relative humidity	○	○	○	○	○	○	○
Lighting	○	○	○	○	○	○	○
Acoustic	○	○	○	○	○	○	○
Overall environment	○	○	○	○	○	○	○

3. **IAQ Satisfaction survey II**

	Very dissatisfied Neutral Very satisfied						
	−3	−2	−1	0	1	2	3
Indoor air quality	○	○	○	○	○	○	○

## References

[1] Klepeis N.E., Nelson W.C., Ott W.R., Robinson J.P., Tsang A.M., Switzer P., Behar J.V., Hern S.C., Engelmann W.H. (2001). The National Human Activity Pattern Survey (NHAPS): A resource for assessing exposure to environmental pollutants. J. Expo. Anal. Environ. Epidemiol..

[2] Duan X., Zhao X., Wang B., Chen Y., Cao S. (2015). Highlights of the Chinese Exposure Factors Handbook (Adults).

[3] Jensen K.L., Toftum J., Friis-Hansen P. (2009). A Bayesian Network approach to the evaluation of building design and its consequences for employee performance and operational costs. Build. Environ..

[4] MacNaughton P., Pegues J., Satish U., Santanam S., Spengler J., Allen J. (2015). Economic, environmental and health implications of enhanced ventilation in office buildings. Int. J. Environ. Res. Public Health.

[5] Wyon D.P. Indoor environmental effects on productivity. Proceedings of the IAQ.

[6] Toftum J., Reimann G., Foldbjerg P., Clausen G., Fanger P.O. Perceived air quality, thermal comfort, and SBS symptoms at low air temperature and increased radiant temperature. Proceedings of the 9th International Conference on Indoor Air Quality and Climate.

[7] Seppänen O., Fisk W.J., Lei Q.H. Room temperature and productivity in office work. Proceedings of the Healthy Buildings 2006 Conference.

[8] Wong L.T., Mui K.W., Hui P.S. (2008). A multivariate-logistic model for acceptance of indoor environmental quality (IEQ) in offices. Build. Environ..

[9] Lan L., Wargocki P., Lian Z. (2011). Quantitative measurement of productivity loss due to thermal discomfort. Energy Build..

[10] Wang D., Xu Y., Liu Y., Wang Y., Jiang J., Wang X., Liu J. (2018). Experimental investigation of the effect of indoor air temperature on students’ learning performance under the summer conditions in China. Build. Environ..

[11] Geng Y., Ji W., Lin B., Zhu Y. (2017). The impact of thermal environment on occupant IEQ perception and productivity. Build. Environ..

[12] Al Horr Y., Arif M., Kaushik A., Mazroei A., Katafygiotou M., Elsarrag E. (2016). Occupant productivity and office indoor environment quality: A review of the literature. Build. Environ..

[13] Dockery D.W., Arden Pope C., Xu X., Spengler J.D., Ware J.H., Fay M.E., Ferris B.G., Speizer F.E. (1993). An association between air pollution and mortality in six US Cities. N. Engl. J. Med..

[14] Zhou Z., Liu Y., Yuan J., Zuo J., Chen G., Xu L., Rameezdeen R. (2016). Indoor PM_2.5_ concentrations in residential buildings during a severely polluted winter: A case study in Tianjin, China. Renew. Sustain. Energy Rev..

[15] Xia T., Chen C. (2017). Differentiating between indoor exposure to PM_2.5_ of indoor and outdoor origin using time-resolved monitoring data. Build. Environ..

[16] Du Y., Wang Y., Du Z., Zhang Y., Xu D., Li T. (2018). Modeling of residential indoor PM_2.5_ exposure in 37 counties in China. Environ. Pollut..

[17] Chamseddine A., Alameddine I., Hatzopoulou M., El-Fadel M. (2019). Seasonal variation of air quality in hospitals with indoor–outdoor correlations. Build. Environ..

[18] Persily A.K. (1997). Evaluating building IAQ and ventilation with indoor carbon dioxide. ASHRAE Trans..

[19] Intergovernmental Panel on Climate Change (2014). Contribution of Working Groups I, II and III to the Fifth Assessment Report of the Intergovernmental Panel on Climate Change. Climate Change 2014: Synthesis Report.

[20] Janssen J. (1989). Ventilation for Acceptable Indoor Air Quality. ASHRAE J..

[21] Petty S. (2014). Summary of ASHRAE’s Position on Carbon Dioxide (CO_2_) Levels in Spaces. http://www.eesinc.cc/downloads/CO2positionpaper.pdf.

[22] Wargocki P., Wyon D.P., Baik Y.K., Clausen G., Fanger P.O. (1999). Perceived air quality, sick building syndrome (SBS) symptoms and productivity in an office with two different pollution loads. Indoor Air..

[23] Wargocki P., Wyon D.P., Fanger P.O. Productivity is affected by the air quality in offices. Proceedings of the Healthy Buildings 2000.

[24] Wyon D.P. (2004). The effects of indoor air quality on performance and productivity. Indoor Air..

[25] Mui K.W., Wong L.T., Hui P.S., Chan W.Y. (2009). Formaldehyde exposure risk in air-conditioned offices of Hong Kong. Build. Serv. Eng. Res. Technol..

[26] Satish U., Mendell M.J., Shekhar K., Hotchi T., Sullivan D. (2012). Concentrations on Human Decision-Making Performance. Environ. Health Perspect..

[27] Vehviläinen T., Lindholm H., Rintamäki H., Pääkkönen R., Hirvonen A., Niemi O., Vinha J. (2016). High indoor CO_2_ concentrations in an office environment increases the transcutaneous CO_2_ level and sleepiness during cognitive work. J. Occup. Environ. Hyg..

[28] Allen J.G., MacNaughton P., Satish U., Santanam S., Vallarino J., Spengler J.D. (2016). Associations of cognitive function scores with carbon dioxide, ventilation, and volatile organic compound exposures in office workers: A controlled exposure study of green and conventional office environments. Environ. Health Perspect..

[29] Ministry of Housing and Urban-Rural Development of the People’s Republic of China (2014). Standard for Lighting Design of Buildings/GB 50034-2013.

[30] World Health Organization (2005). WHO Air Quality Guidelines for Particulate Matter, Ozone, Nitrogen Dioxide and Sulfur Dioxide: Global Update 2005: Summary of Risk Assessment. http://www.who.int/iris/handle/10665/69477.

[31] Ministry of Ecology and Environment of the People’s Republic of China (2012). Ambient Air Quality Standards GB 3095-2012.

[32] Cui X., Li F., Xiang J., Fang L., Chung M.K., Day D.B., Mo J., Weschler C.J., Gong J., He L. (2018). Cardiopulmonary effects of overnight indoor air filtration in healthy non-smoking adults: A double-blind randomized crossover study. Environ. Int..

[33] Flegal K.M., Shepherd J.A., Looker A.C., Graubard B.I., Borrud L.G., Ogden C.L., Harris T.B., Everhart J.E., Schenker N. (2009). Comparisons of percentage body fat, body mass index, waist circumference, and waist-stature ratio in adults. Am. J. Clin. Nutr..

[34] World Health Organization (2017). Global Health Observatory data Tuberculosis, WHO. https://www.who.int/gho/ncd/risk_factors/bmi_text/en/.

[35] Liu H., Liao J., Yang D., Du X., Hu P., Yang Y., Li B. (2014). The response of human thermal perception and skin temperature to step-change transient thermal environments. Build. Environ..

[36] Sanni-Anibire M.O., Hassanain M.A. (2016). Quality assessment of student housing facilities through post-occupancy evaluation. Archit. Eng. Des. Manag..

[37] Huizenga C., Zagreus L., Arens E., Lehrer D. Measuring indoor environmental quality: A web-based occupant satisfaction survey. Proceedings of the Greenbuild 2003.

[38] Barsalou L.W. (1992). Cognitive Psychology an Overview for Cognitive Scientists.

[39] Poremba A. (2015). Neural and behavioral correlates of auditory short-term and recognition memory. Mechanisms of Sensory Working Memory.

[40] Stroop J.R. (1935). Studies of interference in serial verbal reactions. J. Exp. Psychol..

[41] Kagan J., Pearson L. (1966). and Lois Welch, Conceptual Impulsivity and Inductive Reasoning. Child Dev..

[42] Game Clicker or Schulte Tables Rules and Information about the Game. https://brainapps.io/game/Clicker.

[43] Lund A. (2014). Adaptive Attention—Challenge Adjusting Application for Sustained Attention. https://projekter.aau.dk/projekter/files/201270844/Report.pdf.

[44] Shapiro S.S., Wilk M.B. (1968). An analysis of variance test for normality (complete samples). Biometrika.

[45] Kelley K., Preacher K.J. (2012). On effect size. Psychol. Methods.

[46] Miles J., Shevlin M. (2000). Applying Regression and Correlation: A Guide for Students and Researchers.

[47] Zivin J.G., Neidell M. (2012). The impact of pollution on worker productivity. Am. Econ. Rev..

[48] Adhvaryu A., Kala N., Nyshadham A. (2014). Management and Shocks to Worker Productivity: Evidence from Air Pollution Exposure in an Indian Garment. https://economics.sas.upenn.edu/sites/default/files/filevault/event_papers/nyshadham_JMP.pdf.

[49] Chang T., Zivin J.G., Gross T., Neidell M. (2016). Particulate pollution and the productivity of pear packers. Am. Econ. J. Econ. Policy.

[50] Chang T.Y., Zivin J.G., Gross T., Neidell M. (2019). The effect of pollution on worker productivity: Evidence from call center workers in China. Soc. Sci. Electron. Publ..

[51] He J., Liu H., Salvo A. (2019). Severe air pollution and labor productivity: Evidence from industrial towns in China. Am. Econ. J. Appl. Econ..

[52] Paevere P., Brown S., Leaman A., Luther M., Adams R. Indoor environment quality and occupant productivity in the CH_2_ building. Proceedings of the 2008 International Scientific Committee World Sustainable Building Conference.

